# Seronegative Myasthenia Gravis, as a Rare Autoimmune Condition in Turner Syndrome

**DOI:** 10.1155/2017/6863938

**Published:** 2017-11-14

**Authors:** Rimah Sharief, Amir Miodovnik, Roja Motaghedi

**Affiliations:** Infant and Children's Hospital of Brooklyn, Maimonides Medical Center, 977 48th Street, Brooklyn, NY 11219, USA

## Abstract

Girls with Turner syndrome (TS), especially with isochromosome 46,X,i(X)(q10), are prone to develop autoimmunity. Associations of several autoimmune conditions with TS have been frequently described in the past. However, the unique combination of TS and myasthenia gravis (MG) has been reported only once before in a girl with mosaic monosomy 45,X/46,XX. Here, we present the second case of a girl affected with seronegative MG but with mosaic isochromosome TS. This is a child with developmental delay presented with muscle weakness, frequent fall, and bilateral ptosis. Diagnosis of MG was made based on positive Tensilon and electromyography tests and excellent response to intravenous immunoglobulin. At the age of 11 years due to short stature and developmental delay, a karyotype was done and revealed the mosaic isochromosome 45,X/46,X,i(X)(q10). Overall, clinicians should be aware of the vulnerability of girls with TS to autoimmunity, especially if the isochromosome 46,X,i(X)(q10) karyotype is identified. Furthermore, if a child with TS develops muscle weakness, ptosis, or ophthalmoplegia, MG should also be included in the differential diagnosis, particularly if other concurrent autoimmune conditions are present.

## 1. Introduction

Turner syndrome (TS) is caused by total or partial loss or defect of the female second sex chromosome (X) [1]. The reported incidence is approximately 1 in 2000–2500 live births [2, 3], and 1 in 60 cases of female with short stature are affected with TS [2]. Monosomy X (45,X) is the most common overall karyotype, encountered in approximately 45% of diagnosed cases, whereas the most common X chromosome structural defect is the isochromosome 46,X,i(X)(q10) that comprises approximately 15–18% of total cases of TS [3].

The isochromosome 46,X,i(X)(q10) is a duplication of the long arm of the X chromosome, fused at the head with a missing short arm [4]. Other less common structural abnormalities include ring chromosome (r) and deletion of Xp or Xq; both could present as mosaic with another cell line. The structurally defective chromosomes are believed to be fetoprotective, compared to the 45,X karyotype that explains the higher ratio of 46,X,i(X)(q10) and 46,X/r(X) fetuses who are diagnosed as pregnancy advances to term [5].

Depending on the degree of the mosaicism and its timing during development, girls with TS have a series of phenotypic features with various severity [2], most notably short stature and gonadal dysgenesis [1]. Other features include but are not limited to cognitive deficits, skeletal deformities, cardiovascular diseases, hearing difficulties, renal anomalies, and a high propensity to develop an autoimmune condition that is the focus of this report [6].

All girls with various forms of Turner syndrome are at risk for autoimmunity [6], but females with 46,X,i(X)(q10) are specifically at a higher risk [7–10]. The most common autoimmune association is thyroiditis, often in the form of Hashimoto's thyroiditis [7–10], but Graves' disease can also be seen [11]. Elsheikh et al. [10] have reported that approximately 83% of females with 46,X,i(X)(q10) have autoimmune thyroiditis, compared to 45% of females with monosomy X and 24% with mosaic TS. Association of other autoimmune conditions has been summarized in Table 1 and includes celiac disease (CD), inflammatory bowel disease (IBD), and type 1 diabetes mellitus (T1DM) [7, 9, 12]. Vitiligo, alopecia areata, psoriasis [9], primary biliary cirrhosis [13], systemic sclerosis [14], and autoimmune hypophysitis [15] have rarely been reported in girls with TS as well. Even some of the predominantly male-dominant autoimmune conditions, such as Dupuytren's contracture and ankylosing spondylitis, seem to be more common in girls with TS (with about fivefold increase in girls with 46,X,i(X)(q10) karyotype, compared to unaffected females) [16].

Myasthenia gravis (MG) has been documented only once before in a 15-year-old girl with Turner syndrome and 45,X/46,XX karyotype [17]. Here, we present a second case affected with MG but with a mosaic 45,X/46,X,i(X)(q10) Turner syndrome. Clinical comparison of the first case to our patient with respect to MG is presented in Table 2.

## 2. Clinical Presentation

Our patient is a 10-year-old girl of Jamaican descent, a product of nonconsanguineous marriage, who was born premature at 32 weeks of gestation with a birth weight of 4 lb 4 oz/1.928 kg (appropriate for gestational age). Developmental delays and learning difficulties were noted at an early age, requiring intense special education with placement in small, self-contained classroom at the kindergarten level.

At the age of 6 years, she experienced unilateral (left) droopy eyelid and frequent falls, associated with generalized fatigue. Neurological evaluation revealed negative acetylcholine receptor binding and blocking antibodies (AChR-Abs) and muscle-specific kinase antibodies (MuSK-Abs). However, Tensilon test (TT) and electromyography (EMG) were both suggestive of generalized MG, excluding the congenital myasthenic syndromes and other conditions mimicking MG. Diagnosis of seronegative MG was initially considered based on the findings from TT and EMG and, subsequently, confirmed by excellent response to periodic intravenous immunoglobulin (IVIG) infusion (1 g/kg infusion on 2 consecutive days every 4 weeks). In fact, the IVIG was initially administered at every 5th weekly interval, but her symptoms recurred towards the end of the 4th week. This prompted the change of the frequency to every 4th week with significant improvement of droopy eyelid, excessive fatigue, and frequent fall.

At the age of 10 years and 4 months, due to growth deceleration and significant developmental issues, she had a formal multidisciplinary evaluation by a pediatric developmental specialist and an endocrinologist. During the initial endocrine evaluation, she was noted to be short with a height at the 3rd percentile for age and sex, as compared to her midparental height of the 75th percentile. Her physical features were only mildly consistent with TS that likely contributed to delayed diagnosis up to that point. She was noted to have an oblong face with mild facial dysmorphism, down-slanting eyes, bushy eyebrows, low hairline, bilateral in toeing, and her left nipple noted to be laterally displaced. Madelung deformity was not present. In terms of puberty, she had pubic hair development at Tanner stage 2 without thelarche, and biochemically, she was prepubertal (FSH: 8.4 mlU/ml (8.4 IU/L), LH < 0.2 mlU/ml (<0.2 IU/L), and *E*2 < 15 (<4.086 pmol/L)).

Formal developmental evaluation revealed global delays in cognitive, social, adaptive, and motor skills. She was in the 3rd grade but performing well below the grade level. While she was friendly and cooperative during examination, her social interactions appeared immature for her age. There were also concerns regarding hyperactivity, poor concentration, and distractibility, and she met diagnostic criteria for attention-deficit/hyperactivity disorder (ADHD). Overall, her cognitive profile was consistent with other girls who have Turner syndrome (TS). Girls with TS often show selective impairment in nonverbal skills relative to verbal domains on standardized intelligence tests. In addition, they frequently display more difficulty with problem-solving tasks (e.g., mathematics), visuospatial awareness (e.g., difficulty with direction sense), and slower processing speed, with higher rates of ADHD than in the general population [18].

Given her intellectual impairments, short stature, and a mild dysmorphic feature, a genetic workup was carried out. Karyotype with 30 cell counts revealed mosaic Turner syndrome with isochromosome 45,X (23 cells)/46,X,i(X)(q10) (7 cells). In addition, the CGH microarray analysis confirmed the 45,X/46,X,i(X)(q10) karyotype and showed a duplication of 311.5 kb on the chromosome 6q22.33 that seemed to be maternally inherited but of no clinical significance. This diagnosis subsequently triggered cardiac, audiology, and kidney evaluations. A cardiac murmur was present, but echocardiographic examination did not reveal any structural abnormality. Hearing testing was normal initially, but repeated evaluations revealed mild left ear conductive hearing loss. Furthermore, renal ultrasound showed a mild increase in echogenicity in both kidneys, without any functional impairment or significant structural defect. Persistent asymptomatic nonimmune transaminitis and leukopenia, commonly seen in Turner syndrome, were also noted.

Since autoimmunity is common in Turner syndrome and our patient was already affected with MG, we carried out an extensive antibody screening that revealed positive anti-thyroperoxidase antibodies (α-TPO), anti-thyroglobulin antibodies (α-Tg), and anti-glutamic acid decarboxylase-65 antibodies (GAD-65 Ab). Of note, GAD-65 is one of the 2 isoenzymes of GAD that is present in pancreatic islet cells and nerve cell terminals and synapses. In the nervous system, GAD-65 is involved in synthesis and release of γ-aminobutyric acid (GABA), which is the main CNS inhibitory neurotransmitter. GAD-65 positivity has been linked to many CNS disorders such as immune-mediated cerebellar ataxia (IMCA), refractory seizures, and stiff person syndrome (SPS) besides type 1diabetes mellitus (T1DM) [19]. Clinical characteristics of our patient with a complete list of antibodies screened are provided in Table 2.

As presented, our patient and previously reported case by Chen et al. [17] were both diagnosed with autoimmune thyroiditis, but unlike the first case, our patient has remained euthyroid up to this point. Also, the case reported by Chen et al. carried a mosaic 45,X/46,XX karyotype, while our patient is a mosaic 45,X/46,X,i(X)(q10) with negative MG-related antibodies.

### 2.1. Follow-Ups and Interventions

The thyroid function test has been normal up to this point. As mentioned above, she received periodic IVIG infusion for MG with good control.

In terms of neurodevelopmental intervention, she receives intense therapy and special education. For her height, she was immediately started on growth hormone at the dose of 0.35 mg/kg/week and eventually increased to 0.37 mg/kg/week. We plan to start estrogen replacement at the age of 12 years.

## 3. Conclusions and Discussion

MG has been reported only once before [17] in TS, and our patient is the second case (Table 2). As noted above, our case did not test positive for MG-related antibodies and was diagnosed based on clinical findings and response to treatment. However, it is known that autoantibodies (AChR antibodies or MuSK antibodies) could be absent in 10–20% of established cases of MG [20], in this case also known as double seronegative MG. However, the seronegative MG is also an autoimmune condition, but lack of antibody positivity is presumed to be due to an insensitivity of the commercial assay to detect antibodies or presence of antibodies towards other antigen particles [20], in the neuromuscular junction, such as cortactin antibodies [21], low-density lipoprotein receptor-related protein 4 (Lrp4) [22], and anti-striated muscle antibodies (α-SM) not measured by the commercial assay.

It is very well known that children with Turner syndrome, especially TS with 46,X,i(X)(q10), are at high risk of developing autoimmunity with thyroid being the most common target of immune response. The significant tendency of females with TS to develop autoimmunity is not fully understood but presumed to be due to the lack or abnormality of the second X chromosome (X chromosome haploinsufficiency), especially Xp monosomy, which subsequently results in abnormal T-cell regulatory function and inability to develop self-tolerance in early life [9]. In addition, a dysregulation of immune response with shift to proinflammatory cytokine response (IL6 and TGF β2) and decrease in the anti-inflammatory cytokines (IL10 and TGF β1) has also been observed in females with TS and postulated to play a role in tendency to develop self-antibody [9].

In conclusion, clinicians need to be aware of this propensity and have high index of suspicion for a range of autoimmune conditions, especially when the genetic defect is isochromosome 46,X,i(X)(q10). Furthermore, myasthenia gravis should be in mind, when a child with Turner syndrome presents with muscle weakness, droopy eyelid, or ophthalmoplegia, even though an isolated ptosis is also a known feature of girls with TS [6].

## Figures and Tables

**Table 1:** Estimated prevalence of autoimmunity in Turner syndrome.

Autoimmunity	Prevalence in TS (% of diagnosed cases)	Author, year (ref.)
Hashimoto's thyroiditis	30–60	Elsheikh et al., 2001 [10]

Celiac disease	2.7–17	Bakalov and Gutin, 2012 [9]
		Mortensen et al., 2009 [12]

IBD	4	Bakalov and Gutin, 2012 [9]

Type 1 diabetes mellitus	0.9–4	Bakalov and Gutin, 2012 [9]
		Mortensen et al., 2009 [12]

Psoriasis	3.1	Bakalov and Gutin, 2012 [9]

Graves' disease	0.7–2.7	Elsheikh et al., 2001 [10]
		Valenzise et al., 2014 [11]

Adrenal antibodies	1-2	Hamza et al., 2013 [8]
		Mortensen et al., 2009 [12]

JRA and RA	0.9	Bakalov and Gutin, 2012 [9]

Ankylosing spondylitis	0.4	Bakalov and Gutin, 2012 [9]

IBD: inflammatory bowel disease; JRA: juvenile rheumatoid arthritis; RA: rheumatoid arthritis.

**Table 2:** Clinical characteristics of our patient compared to the first reported case of TS affected with MG.

Clinical characteristics	Our index case	First published case^1^ (Chen et al., 1978) [17]
Age at MG onset	6 years	15 years

Age of TS diagnosis	10 years	10 years

Ethnicity	African American	Caucasian

Genetic defects (TS karyotype)	45,X/46,X,i(X)(q10)	45,X/46,XX

MG symptoms	Left eye ptosis (+), ophthalmoplegia (−)	Bilateral ptosis (+), ophthalmoplegia (+)
	Frequent fall (+), fatigue (+)	Frequent fall (+), fatigue (+)

Serology	Negative AChR, MuSK, and α-SM Abs	AChR Abs and MuSK not performed. Negative α-SM Abs

Tensilon test and EMG	Positive	Positive

Thyroid autoimmunity	α-TPO (+) and α-Tg (+)	α-TPO (+) and α-Tg (+)
	Euthyroid up to this point	Developed hypothyroid at the age of 15 y

Other antibody screening	*Positive*: α-GAD-65	*Positive*: ASMA, α-myelin Ab
	*Negative*: AGA, ASMA, α-SM Ab, ANA, α-dsDNA, α-ssDNA, α-MPO, proteinase-3, α-adrenal Abs, direct α-globulin Abs	*Negative*: α-DNA Ab, α-adrenal Ab, α-Coomb^2^ Ab

Initial treatment	Cholinesterase inhibitors and glucocorticoid with no response	Cholinesterase inhibitors with no response

Definitive treatment	Periodic IVIG infusion	Thymectomy followed by cholinesterase inhibitors

EMG: electromyography; AChR-Abs: acetylcholine receptor antibodies; α-TPO: anti-thyroperoxidase; α-Tg: anti-thyroglobulin; α-GAD-65: anti-glutamic acid decarboxylase-65; ASMA: anti-smooth muscle antibodies; α-SM Ab: anti-striated muscle antibodies; ANA: antinuclear antibodies; MPO: myeloperoxidase; AGA: anti-gliadin antibodies. ^1^All the information in the 3rd column was obtained from the first published case for comparison purpose. ^2^Direct antiglobulin Ab test (direct Coomb test) was done to evaluate for hemolytic anemia. Chen et al. did not mention which antibodies were tested.

## Abbreviations

TS: Turner syndrome
46,X,i(X)(q10): Isochromosome Xq
MG: Myasthenia gravis
AChR-Abs: Acetylcholine receptor antibodies
α-GAD-65: Anti-glutamic acid decarboxylase-65.
